# Natural peste des petits ruminants virus infection in Black Bengal goats: virological, pathological and immunohistochemical investigation

**DOI:** 10.1186/s12917-014-0263-y

**Published:** 2014-11-14

**Authors:** Emdadul Haque Chowdhury, Ataur Rahman Bhuiyan, Mohammad Mushfiqur Rahman, Mohammad Sahinur Alam Siddique, Mohammad Rafiqul Islam

**Affiliations:** Department of Pathology, Faculty of Veterinary Science, Bangladesh Agricultural University, Mymensingh, Bangladesh; Department of Livestock Services, Dhaka, Bangladesh

**Keywords:** PPR, Natural outbreak, Pathology, Antigen detection, Nucleic acid detection

## Abstract

**Background:**

Peste des Petits Ruminants (PPR), also known as Goat Plague, occurs in goats, sheep and related species. It is caused by a *morbillivirus* in the family *Paramyxoviridae.* In Bangladesh PPR is endemic and it causes serious economic losses. Pathology of PPR has been reported in different goat and sheep breeds from natural and experimental infections. Field results are better indicators of pathogenicity of the circulating virus. The severity of the disease varies with species, breed and immune status of the host. Pathological investigations of natural outbreaks of PPR in Balck Bengal goats are very limited. The current investigation was aimed at describing pathology and antigen localization in natural PPR infections in Black Bengal goats.

**Results:**

A total of 28 outbreaks were investigated clinically and virologically. Average flock morbidity and mortality were 75% and 59%, respectively, with case fatality rate of 74%. Necropsy was conducted on 21 goats from 15 outbreaks. The major gross lesions were congestion of gastrointestinal tract, pneumonia, engorged spleen, and oedematous lymphnodes. Histopathological examination revealed severe enteritis with denudation of intestinal epithelium, severe broncho-interstitial pneumonia with macrophages within lung alveoli and extensive haemorrhages with depletion of lymphoid cells and infiltration of macrophages in the sinuses of spleen. In lymph nodes, the cortical nodules were replaced by wide sinusoids with severe depletion of lymphocytes, infiltration of mononuclear cells and some giant cells in sub-capsular areas and medullary sinuses. PPR virus antigen was found in pneumocytes and alveolar macrophages in lungs. Viral RNA could be detected by RT-PCR in 69 out of 84 nasal swab, 59 out of 84 blood and 21 out of 21 lymph node samples. Sequence analyses revealed closeness of Bangladeshi strains with other recent Asian isolates.

**Conclusion:**

Natural outbreaks of PPR in Black Bengal goats in Bangladesh resulted in 75% and 59% flock morbidity and mortality, respectively, with a case fatality rate of 74%. The striking histo-morphologic diagnosis of PPR was acute pneumonia and severe gastro-enteritis. A detailed experimental pathological study on Black Bengal goats infected with recent isolates is required.

## Background

Peste des Petits Ruminants (PPR), also known as Goat Plague and Ovine Rinderpest, occurs in goats, sheep and related species. It is caused by a *morbillivirus* in the family *Paramyxoviridae.* It is closely related to Rinderpest, measles, canine distemper and morbilliviruses of marine mammals and is the most economically important viral disease of small ruminants [1-3]. In the field, PPR virus causes disease in goats and sheep. Goats are usually considered to be more susceptible than sheep, but this is not always the case; cattle and pigs can be infected sub-clinically by experimental infections [4,5]). The disease is characterized by high fever, discharges from nose, eyes and mouth; profuse diarrhoea; pneumonia; and oral erosion, with high morbidity [6-8]. In susceptible populations, morbidity and mortality rate are 5-90% and 50-80%, respectively [9-11]. Post mortem examination reveals frothy exudates in respiratory tract, congestion and partial consolidation of lungs, congested mesenteric and bronchial lymph nodes, severe congestion, haemorrhages and edema in gastro-intestinal tract [6,12-14]. Histologically characteristic lesions are usually seen in digestive and respiratory systems [15]. In intestine histologic findings of natural PPR virus infection include atrophic villi with partial denudation of epithelial lining and intense diffusion of mononuclear cells in the lamina propria and sub-mucosa [16,17]. The lungs show broncho-interstitial pneumonia characterized by proliferation of bronchiolar lining epithelium, intense diffusion of mononuclear cells mainly in lymphoid, macrophages and plasma cells in the pericardial, the interstitial tissue and alveolar lumina [17]. In Bangladesh, the first outbreak of PPR was recorded in 1993 [18]. Since then outbreaks are being reported regularly across the country. In 2010, there were 84,000 veterinary clinic cases, causing an estimated loss of Taka: 1,842 million (US$ 24.6 million) [19], but the actual number of cases may be even more because infected cases from remote rural areas were not brought to the veterinary clinic. Although prevention is the main strategy against viral diseases, supportive treatment may prevent some loss. Knowledge of pathology and pathogenesis may help in formulation of supportive treatment. Pathology of PPR has been reported in different goat and sheep breeds from experimental infections [6,16,17,20]. Pathological data from field cases are very important as they could better characterize the pathogenicity of the circulating virus. The severity of the disease varies with species, immunity and breed of the host [21]. Pathological investigations of natural outbreaks of PPR in Black Bengal goats are very limited [18].The current investigation was aimed at describing the pathology and antigen localization in natural PPR infections in Black Bengal goats.

## Results

### Clinical findings

This investigation examined 28 outbreaks of PPR in Black Bengal goats from different parts of the country having average 75% (63% to 100%) morbidity, 59% (23% to 100%) mortality and 74% (26% -100%) case fatality (Table 1). Mortality was higher in the young (5–12 month of age) goats (field veterinarian’s observation). The veterinarians treated 250 – 300 out of 947 affected goats with antibiotic, antihistamine, astringent and 5% dextrose saline for a week. A few (±30) of the treated goats survived after treatment but 5 goats died after a month due to secondary bacterial infections (field veterinarian’s report, data not shown). The goat that died after a month was not included in the mortality calculation. The most frequently observed signs were abrupt rise of body temperature (105-106°F) that lasted for 3 to 5 days. The affected animals were severely depressed, standing apart and had impaired appetite and constipation. Rise of body temperature was accompanied by watery discharges (nasal, ocular and oral), which lasted for 4 to 5 days. Very often ocular discharges were associated with conjunctivitis; nasal discharge became purulent and blocked nostril in advanced stages. Medial canthus revealed some crusts at later stages. Small areas of necrosis were seen on the visible nasal mucous membranes. The erosive and necrotic stomatitis was seen on hyperaemic gums, cheeks, dental pad and on tongue, with frothy salivation in some cases. In some cases circular raised non-bleeding lesions were present on the tongue. Lesions similar to orf developed at mucocutaneous junction of mouth in many animals at later stages of the disease. Abortion was frequent at all stages of pregnancy. Diarrhoea was common with sub-normal body temperature usually 5 to 6 days after the first appearance of clinical signs. The hindquarters of diarrhoeic goats were soiled with liquid greenish feces. Bronchopneumonia, evidenced by coughing, was common at the later stages of PPR. In severe cases, most animals died within a week. A subnormal temperature preceded death in animals when severe diarrhoea continued for a few days.

**Table 1:** **Morbidity and mortality of goats in different outbreaks of PPR in Bangladesh during May 2008 to June 2010**

**Outbreaks**	**Location**	**Breed**	**Flock size**	**Age (m)**	**No. of goats affected**	**No. of goats died**	**Flock Morbidity (%)**	**Flock Mortality (%)**	**Case fatality (%)**	**No. of necropsy**
1	Chittagong	BB	16	5-30	11	10	69	63	91	2
2	Narayangong	BB	47	2-36	47	41	100	87	87	nd
3	Rajshahi	BB	22	1-36	22	18	100	82	82	2
4	Dhaka	BB	7	6-24	6	4	86	57	67	1
5	Narayangong	BB	6	6-8	5	4	83	67	80	2
6	Chittagong	BB	8	5-24	5	2	63	25	40	nd
7	Sylhet	BB	6	6-30	6	3	100	50	50	1
8	Dhaka	JP	31	1-36	27	7	87	23	26	nd
9	Dhaka	BB	8	6-8	8	8	100	100	100	1
10	Sylhet	BB	6	6-24	5	4	83	67	80	nd
11	Mymensingh	BB	6	6-24	5	4	83	67	80	1
12	Dhaka	JP	12	5-30	12	9	100	75	75	nd
13	Dhaka	BB	69	1-36	60	58	88	85	97	nd
14	Sathkira	BB	805	1-36	540	425	67	53	79	nd
15	Jessore	JP	7	6-24	6	4	86	57	67	nd
16	Mymensingh	BB	25	8-12	23	19	92	76	83	nd
17	Dhaka	BB	22	1-36	22	16	100	73	73	2
18	Mymensingh	BB	22	3-36	17	11	77	50	65	2
19	Dhaka	BB	15	8-24	12	10	80	67	83	1
20	Dhaka	JP	15	5-24	13	11	87	73	85	nd
21	Jessore	BB	8	4-24	6	4	75	50	67	1
22	Dhaka	JP	12	5-24	11	6	92	50	55	nd
23	Rajshahi	BB	12	5-30	8	6	67	50	75	nd
24	Dhaka	BB	8	5-30	6	4	75	50	67	1
25	Rajshahi	BB	6	8-15	6	6	100	100	100	1
26	Mymensingh	BB	6	6-15	4	3	67	50	75	2
27	Narayangong	BB	7	6-30	6	4	86	57	67	1
28	Jessore	BB	50	1-36	48	41	96	82	85	nd
Total	1264		947	742	74.92	58.71	74.32	21		

BB: Black Bengal Goat, JP: Jamuna Pari, m: month, nd: Not done, only clinical history and nasal swabs and/or blood smeared filter papers were received from the veterinary surgeons for diagnosis.

### Necropsy findings

**Figure 1:**
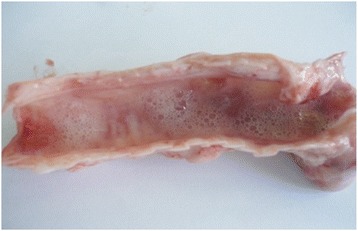
**Necropsy finding of trachea of a PPR virus infected goat.** Trachea revealed severe congestion in tracheal mucous membrane with frothy exudates in the lumen.

**Figure 2:**
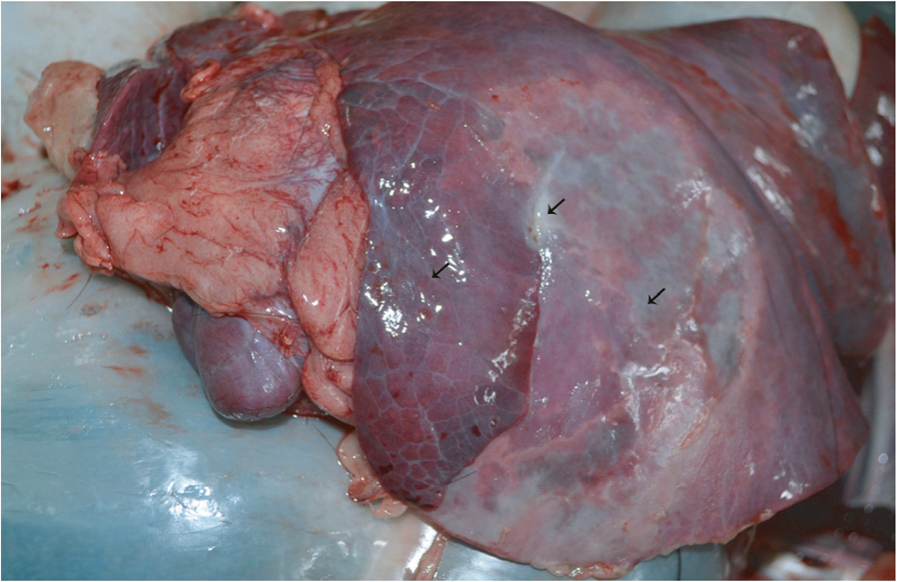
**Necropsy finding of lung of a PPR virus infected goat.** Lung showed pneumonia with consolidation and accumulation of fibrin (arrow) over the surface of the lung. Lung showed muscular appearance.

**Figure 3:**
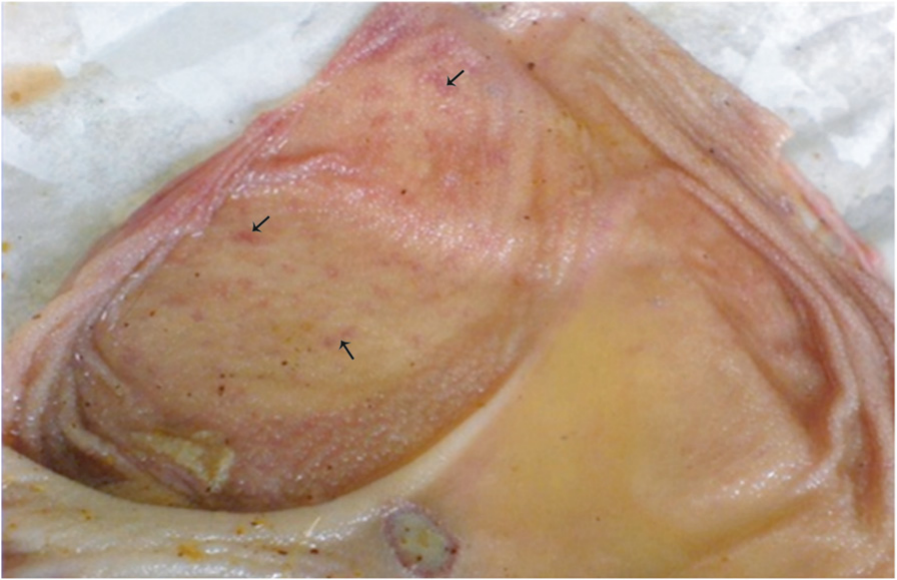
**Necropsy finding of rumen of a PPR virus infected goat.** Ecchymotic haemorrhages (arrow) were present on the mucosal surface of rumen.

**Figure 4:**
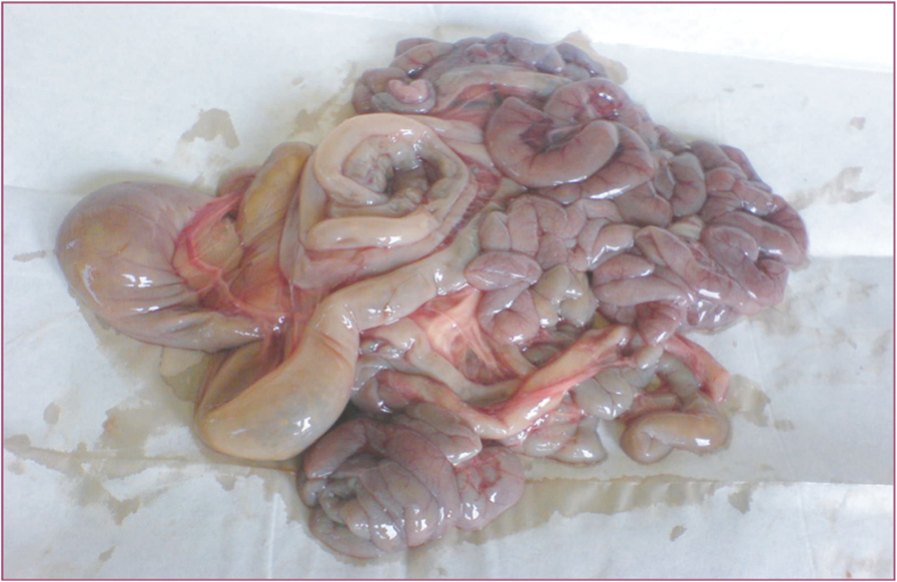
**Necropsy finding of intestine of a PPR virus infected goat.** Severe enteritis with haemorrhages and congestion was present in small and large intestine. The intestine contained diarrheic faeces and gas.

**Figure 5:**
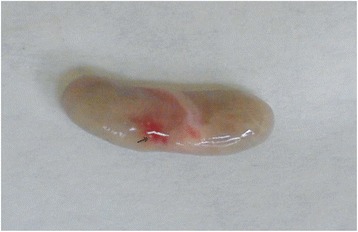
**Mesenteric lymph node of a PPR virus infected goat.** Lymph node was oedematous, congested (arrow) and enlarged.

**Figure 6:**
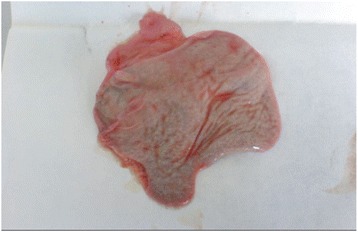
**Spleen of a PPR virus infected goat.** Severe spleen atrophy was present in a goat that died after 15 days of onset of clinical signs of PPR. The nasal swab was positive for PPR virus excretion.

The carcasses were dehydrated and emaciated with sunken eyes, but some goats were in good bodily condition after death at early infection. The lesions of respiratory tract included necrotic areas on the mucosa of nostrils and turbinate and severely congested tracheal mucous membrane with white frothy mucus in lumen (Figure 1). The lungs were dark red or purple with areas firm to touch, mainly in the anterior and cardiac lobes (Figure 2). In the digestive tract, rumens of three goats revealed ecchymotic haemorrhages (Figure 3) and streaks of haemorrhages were also seen in duodenum and the terminal ileum (Figure 4). The lymph nodes, especially from the mesentery were severely oedematous, congested and enlarged (Figure 5). The large intestine was usually more severely affected with congestion around the ileocaecal valve, at the caeco-colic junction, and in the rectum, although not in all the carcasses. Spleen was atrophied in cases that died after a couple of weeks (Figure 6). Erosive vulvo-vaginitis was observed in eight animals.

### Histopathological findings

**Figure 7:**
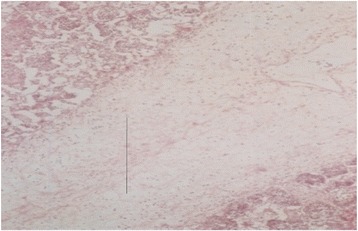
**Histological section of a lung of a PPR infected goat.** Accumulation of fibrin (line) and infiltration of inflammatory cells in the lung parenchyma, H&E X 82.5.

**Figure 8:**
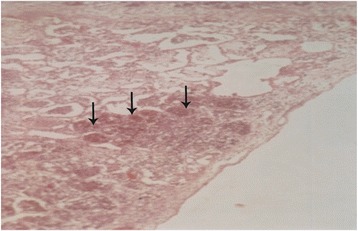
**Histological section of a lung of a PPR infected goat.** Lung alveoli filled with severe infiltration of inflammatory cells (arrow), H & E X 82.5.

**Figure 9:**
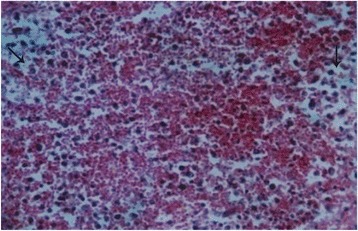
**Histological section of spleen of a PPR infected goat.** Spleen showed accumulation of extravagated Erythrocytes in the pulp and depletion of lymphocytes (arrow). H & E X 82.5.

**Figure 10:**
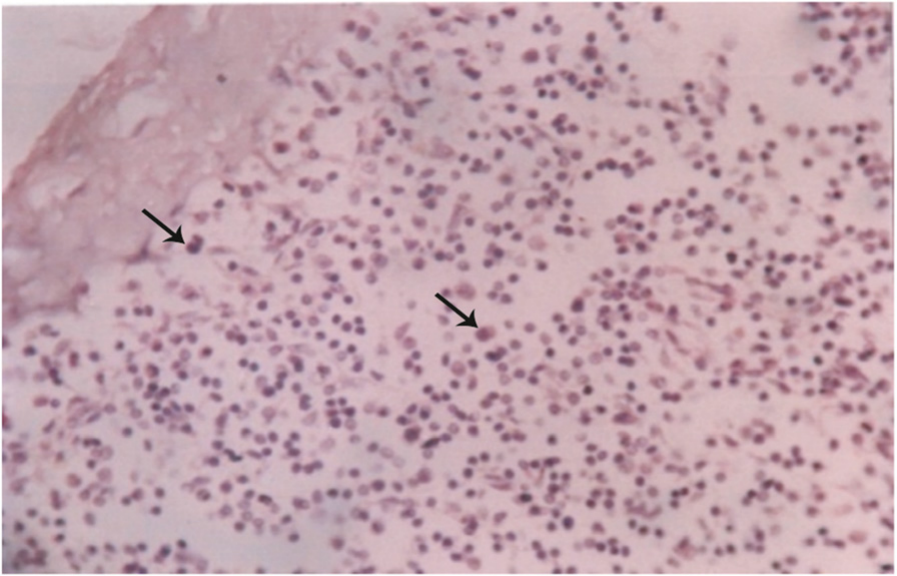
**Histological section of Lymph node of a PPR infected goat.** Lymph node revealed wide sinusoidal depletion of lymphocytes and proliferation of mononuclear cells (arrow). H & E X 330.

Details of the histopathological findings are listed in Table 2. The most remarkable lesions were found in respiratory, alimentary and lymphatic systems. Goats that died within 48 hours of the onset of clinical signs were mostly haemorrhagic and showed severe exudation in the upper respiratory tract and in the lung parenchyma. The exudates in the lung parenchyma often proceeded to fibrinous organization (Figure 7). In goats that died after 3–5 days, lesions of lungs were more severe, with additional involvement of alimentary tract. The lung parenchyma was infiltrated with large mononuclear cells and clumps of these cells were found in the lung alveoli (Figure 8). In some places, alveoli lost its lining of squamous epithelium, which were partially replaced by cuboidal type epithelial cells. The entire alimentary tract revealed haemorrhagic gastroenteritis with infiltration of mononuclear cells in the lamina propria and loss of laminar epithelia. Clumps of epithelial cells were aggregated in the lumen of the intestine. Liver was severely congested and haemorrhagic. Changes in the lymphatic systems appeared more haemorrhagic in goats that died within 2–3 days. Lymphatic organs were depleted of lymphocytes (Figure 9). These lesions were more severe in goats that survived more than a week. Conspicuous lymphoid depletion was seen in Peyer’s patches in 6 cases, in lymph nodes in 7 cases, and in spleen (Figure 10) in 7 cases.

**Table 2:** **Histopathological findings in goats naturally infected with PPR virus**

**Carcass no**	**Lungs**					**Gastro intestinal changes**		**Lymphoid organs (depletion and necrosis)**		**Remarks**
	**Congestion**	**Proliferative changes in alveoli**	**Bronchitis/bronchiolitis**	**Presence of syncytia**	**Sero-fibrinous exudates**	**Desquamation and inflammation**	**Depletion in Payers patches**	**MLN**	**Spleen**	
1	+	+	+	-	-	++	+	+	+	**
2	+++	-	++	-	+	-	-	-	-	*
3	+	+	+	-	-	+	+	+	+	***
4	++	-	+	-	-	-	-	+/−	+	*
5	+	+	+	-	-	+	+	-	-	**
6	+++	-	++	+	+	+	-	-	-	*
7	++	-	+	-	-	+	+	-	-	*
8	+	+	+	+	-	-	+	+	++	***
9	++	-	++	-	+	++	-	+	+	**
10	+	-	+	-	-	++	-	-	-	**
11	+	+	+	+	-	+	+	+	+	***
12	++		+	-	-	++	-	-	-	**
13	+	+	+	+	-	-	-	-	-	*
14	+	-	+	-	-	++	-	+	+	**
15	+	-	+	-	-	+	-	-	-	**

*Died within 24–48 hours, **= 3 – 5 days, ***Died after 7 days, MLN: Mesenteric lymph node, + = visible, ++ = prominent, +++ = severe.

### Localization of antigen

**Figure 11:**
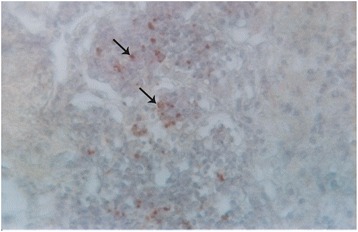
**Immunochemical staining of lung tissues of a PPR infected goat.** PPR virus antigen was detected in the macrophages accumulated in the lung alveoli (arrow) and epithelial cell of lung alveoli, counterstained with hematoxylin, x 82.5.

**Figure 12:**
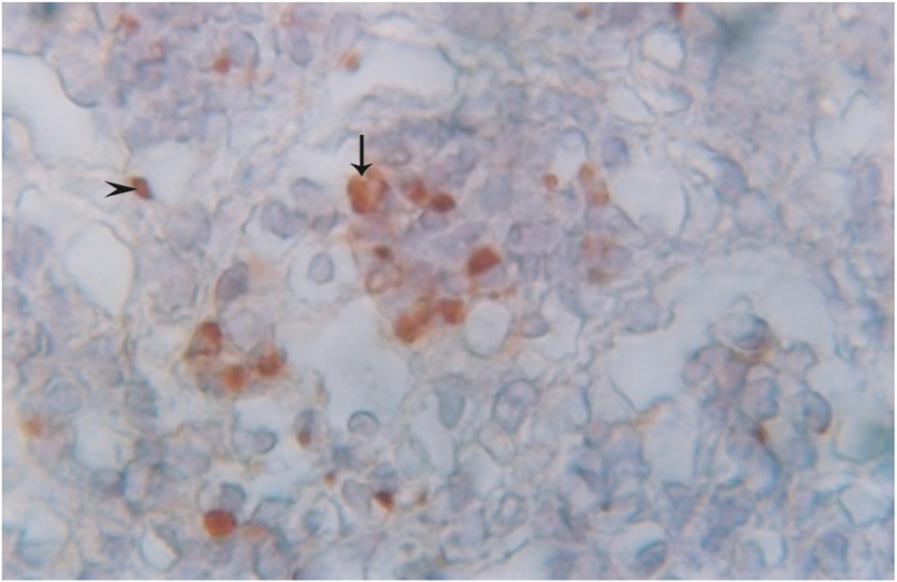
**Immunochemical staining of lung tissues of a PPR infected goat.** Higher magnification of Figure 11 that showed PPR virus antigen in the alveolar epithelial cells (arrow head) and macrophages in alveolar lumen (arrow), counterstained with hematoxylin, x 330.

Only lung tissue was positive for PPR virus antigen. Specific staining was characterized by a diffuse red granular reaction in the cytoplasm and nucleus, often coalescing with each other. specifically antigen was localized in the cytoplasm and nuclei of alveolar and bronchiolar epithelia, macrophages and syncytia (Figures 11 and 12). Other organs were also analyzed but positive reaction was not detected.

### Viral nucleic acid detection in lymph node, blood and nasal swabs

All samples tested from 28 outbreaks yielded PCR products of 448 bp (F gene) and 351 bp (N gene) as stated by Forsyth and Barrett (1995) [22] for PPR virus. Details of the samples are given in Table 3. All of the 21 lymph node homogenates were found positive from 15 outbreaks, where either blood or nasal swabs or both were positive from 13 outbreaks. Viral RNA could be detected in 69 out of 84 nasal swabs and 59 out of 84 blood samples (Table 3). Lymphnode samples were not available from all outbreaks. All the three nasal swab samples from one outbreak were positive but not the blood samples (outbreak no. 20). On the contrary, in another outbreak all the three blood samples were positive but not the nasal swabs (outbreak no. 4). Goats which were positive with nasal swab but not with blood were sampled at the non-febrile stage (diarrhoeic phase) 7 days after the onset of clinical signs. The animal that was positive with blood but negative with nasal swab were found dead at morning within 24 hours of onset of high temperature (107°F).

**Table 3:** **Samples tested from different outbreaks of PPR in Black Bengal goats**

**Outbreaks**	**Location**	**No. samples tested**			**No. of samples positive by RT-PCR**		
		**NSF**	**BSF**	**LH**	**NSF**	**BSF**	**LH**
1	Chittagong	3	3	2	3	2	2
2	Narayangong	3	3	na	3	2	
3	Rajshahi	3	3	2	3	3	2
4	Dhaka	3	3	1	0	3	1
5	Narayangong	3	3	2	2	2	2
6	Chittagong	3	3	na	2	2	
7	Sylhet	3	3	1	3	3	1
8	Dhaka	3	3	na	3	2	
9	Dhaka	3	3	1	2	1	1
10	Sylhet	3	3	na	3	1	
11	Mymensingh	3	3	1	3	2	1
12	Dhaka	3	3	na	1	2	
13	Dhaka	3	3	na	2	2	
14	Sathkira	3	3	na	3	1	
15	Jessore	3	3	na	3	2	
16	Mymensingh	3	3	na	2	1	
17	Dhaka	3	3	2	2	2	2
18	Mymensingh	3	3	2	3	2	2
19	Dhaka	3	3	1	3	2	1
20	Dhaka	3	3	na	3	0	
21	Jessore	3	3	1	2	1	1
22	Dhaka	3	3	na	2	2	
23	Rajshahi	3	3	na	3	2	
24	Dhaka	3	3	1	2	3	1
25	Rajshahi	3	3	1	3	2	1
26	Mymensingh	3	3	2	3	2	2
27	Narayangong	3	3	1	2	2	1
28	Jessore	3	3	na	3	2	
Total	84	84	21	67 (79%)	59 (70%)	21 (100%)	

NSF: nasal swab smeared filter paper, BSF: Blood smeared filter paper, LH: Lymph node homogenates, na: not available, NSF and BSF samples originated from the same goats.

### Sequence analyses

The representative PCR products of F and N genes of one isolate were sequenced from a commercial source and confirmed by comparing with known PPR sequence. The sequences have been submitted to the GenBank [GenBank Accession No. HQ898003 & JF276436]. On phylogenetic analysis the present Bangladeshi strain clustered in Lineage IV and formed a sub-cluster along with recent isolates from Tibet, Tajikistan and India. These isolates have two unique amino acid substitutions (K423Q and E426G) in N protein, which were not evident in other Lineage IV isolates.

## Discussion

Peste des petits ruminant virus (PPRV) produced severe pathological lesions in respiratory tract, alimentary tract and lymphatic system in natural outbreaks in Black Bengal goats. PPR has been one of the most prevalent infectious diseases among goats in Bangladesh and is considered an emerging economically important disease. In the present study, 28 outbreaks in flocks having 1264 goats were investigated. The suspected samples were first screened by RT-PCR for PPR virus. The flock morbidity and mortality in this study were 63 to 100% and 23 – 100%, respectively, whereas few other authors found 5-90% and 50 – 80%, respectively [9-11]. According to field veterinarian’s observations, young goats (6–12 months) were more susceptible than the aged, which are inconsistent with the findings of Taylor et al. [11]. This may be due to maternally derived PPR antibody that persists up to 4 months [23]. It is likely that antibody derived from sub-clinical infection in aged goats protected themselves as well as their young progenies. This observation was in accordance with those reported by Taylor et al. [24]. Some authors reported that more severe disease results from mixed infection of bacteria and viruses [17]. We did not take any attempt to isolate bacteria from these cases. In Bangladesh, when a goat is affected by PPR, veterinarians generally treat with antibiotic, antihistamine, dextrose saline and astringent mixtures. Treatment may help to reduce mortality from PPR due to (i) control of respiratory bacterial infections and (ii) counteracting shock by astringent mixture, dextrose saline and anti-histamine. Some goats that survived the acute stage of the disease died later, possibly due to immunosuppression induced by the PPRV. PPRV can cause severe lymphocytolysis in lymphoid tissues, such as tonsils, spleen, Peyer’s patches, and mediastinal and mesenteric lymph nodes resulting in immunodeficiency [25-27]. Our observation also revealed lymphoid depletion in the lymphatic nodules and spleen.

In the present study in Black Bengal goats, characteristic erosive ulcerative lesions in the mouth and inflammatory lesions in the respiratory and gastrointestinal tract corroborate the findings of previous studies in sheep and other breeds of goats [6-8,12-17,27]. Cast of intra-luminal epithelial cells and macrophages in the lung tissue is a characteristic feature of morbillivirus infection. Immunohistochemically, PPRV were detected in the intra-luminal epithelial tissues and macrophages.

Histologically, proliferation of macrophages in different organs was seen, which indicates primarily non-purulent inflammation. Depletion of lymphocytes from lymph nodes and spleen may be due to affinity of the virus to lymphoid tissue and the involvement of lymphocytes in the pathogenesis of PPR [12]. The virus localizes in the lung parenchyma and causes diffuse interstitial pneumonia with eosinophilic intranuclear inclusion bodies in the pneumocytes [16]. Although we did not find intra-nuclear inclusion bodies with H&E, antigen was detected in pneumocytes and macrophages with immunohistochemistry. Brown et al. [28] used lung tissue for indirect immuno-histochemical methods and detected viral antigen in pneumocytes and alveolar macrophages. We were not able to detect PPRV antigen in tissues of other organs, the reason was not clear to us.

In this investigation, both blood and nasal swab samples were positive for virus in most cases. But 3 goats sampled at non-febrile stage 7 days after onset of clinical signs were positive only with nasal swabs. The febrile stage usually persists for 2 – 3 days and then the virus localizes in the respiratory and associated tissues and therefore excretes the virus through nasal secretion for longer period. Therefore the nasal swab can be a good choice for the PCR detection of PPR virus in infected animals both in febrile and non-febrile stages of the disease.

## Conclusion

The striking histo-morphological diagnosis was acute pneumonia and severe gastro-enteritis. Since Bangladesh is a PPR endemic country, stamping out is not practiced. Therefore, early supportive treatment based on the pathological findings, could reduce the mortality of PPR virus infected goats which is economic for the poor farmer. A detailed experimental pathological study of Black Bengal goats infected with different isolates is required.

## Methods

### Samples

A total of 28 PPR outbreaks from different parts of the country during December, 2008 – December, 2010 were included in this study (Table 1). The size of the affected flocks varied from 6 to 805 with a total of 1264 goats (Table 1). All the farms were in contact with veterinarians, who observed daily for morbidity, mortality and clinical signs and recorded body temperature of the affected goats. The veterinarians were in contact with the investigators and filled in the prescribed clinical examination form. From 15 outbreaks 21 dead goats were subjected to necropsy at the necropsy room of veterinary clinics or at the Department of Pathology, Bangladesh Agricultural University, Mymensingh. From the remaining 13 outbreaks, only nasal and blood smeared filter papers were received from the veterinarians with completed clinical examination form. The blood samples were collected from jugular vein by aseptic means. A few drops of blood were poured on the filter paper, from the base towards the tip, until it was completely soaked. Nasal swabs were collected with sterilized swab sticks, which were smeared on the filter paper. Smeared filter papers were air-dried avoiding direct sunlight and preserved in labelled sterilized Eppendorf tube. The papers and tissues were stored at −70°C at the laboratory until analysis.

### Necropsy

Necropsies were performed on 21 goats of 15 outbreaks that were found dead at farms. None of them received any supportive treatment prior to death. These goats died 24 hours to 7 days after onset of clinical signs. Tissue samples from various organs were collected at necropsy and fixed in 10% neutral buffered formalin. Tissues from bronchial lymph node of dead animals were also collected aseptically for detection of viral RNA by RT-PCR.

### Histopathology

Histopathology was performed on one goat from each of the 15 outbreaks, where necropsy was conducted. Formalin-fixed samples were processed and stained as described [29]. Briefly, the fixed tissues were trimmed and further fixed for 24 hrs. Tissues were kept in running tap water overnight to wash out formalin. The tissues were dehydrated in ascending grades of alcohol (50%, 70%, 80%, 95%) and three changes of absolute alcohol for one hour in each. Sections were cleared in chloroform by two changes, 1½ hours for each. The samples were embedded with molten paraffin wax at 56°C; two changes, 1½ hours for each, and then paraffin blocks were prepared using a template. The tissues were sectioned with a microtome at 5-μm thickness. The sections were allowed to spread on warm water bath (37°C) and taken on grease-free glass slides. A small amount of gelatin was added to the water bath for better adhesion of the section to the slide. The slides were air-dried and kept cool until staining.

The sectioned tissues were deparaffinized in three changes of xylene (three minutes in each) and rehydrated through descending grades of alcohol (three changes in absolute alcohol, three minutes in each; 95% alcohol for two minutes; 80% alcohol for two minutes; 70% alcohol for two minutes) followed by distilled water for five minutes. The tissues were stained with Harris haematoxylin (MERCK, Germany) for fifteen minutes followed by washing in running tap water for 10–15 minutes. The tissues were differentiated in acid alcohol by 2 to 4 dips (1 part HCl and 99 parts 70% alcohol), washed in tap water for five minutes followed by 2–4 dips in ammonia water until sections were bright blue, followed by staining with eosin for one minute. Tissues were dehydrated in alcohol (95% alcohol: three changes, 2–4 dips each; absolute alcohol: three changes 2–3 minutes for each), and cleaned in xylene, three changes (five minutes each). Finally, the sections were mounted with cover slip using DPX (LOBA Chemie, India) and photographed using photo-micrographic camera (Olympus PM-C 35 Model).

### Immunohistochemistry

The tissues were sectioned with a microtome at 5 μm thickness. Sections were allowed to spread on warm water bath (37°C) and taken on poly-L-lysine-coated slides (Thermoshandon). Afterwards the procedure was as follows:

(i) The sections were deparaffinized in fresh xylene (four changes, 5 minutes in each) and rehydrated through descending grades of alcohol (three changes in absolute alcohol, three minutes in each; 95% alcohol for 3 min; 80% alcohol for 3 min; 70% alcohol for 3 min and 50% for 3 min) followed by washing in running tap water for 20 minutes and in distilled water for 5 min. (ii) Sections were placed in a Coplin Jar containing 0.1% trypsin (Sigma-Aldrich) in Tris buffer, pH 7.8 (10 mM) and incubated for 10 minutes at 37°C in water bath, followed by washing in chilled PBS (0.01 M) in another Coplin jar (2 changes, 5 min in each). (iii) The trypsinized sections were fixed in air-free disposable cover plates and stacked in a immunostaining rack (Thermo Shandon). (iv) Endogenous tissue peroxidase was inactivated by applying 0.3% H_2_O_2_ to the sections at room temperature for 10 minutes followed by washing in PBS. (v) The sections were incubated with 100 μl blocking solution (non-immune goat serum) for 10 min, and washed again in PBS for 10 minute. (vi) The sections were incubated with primary antibody 1: 50 dilution of mAb (clone 38–4) against N protein overnight at 4°C in moist chamber (mAb was a kind gift from PPR Reference Laboratory, CIRAD France). (vii) After incubation with primary antibody, the sections were washed in PBS (three washes, each for 3 minutes). (viii) Remaining reaction was developed according to instruction of Histostain® plus Kits (LAB-SA Detection System, Invitrogen). (ix) The reaction was visualized by adding AEC (3-Amino-ethylcarbazole), 3 drops per section with incubation for 10 minutes. The sections were counter-stained with Meyer’s haematoxylin for 5 minutes; excess haematoxylin was removed by washing in running tap water for 10 min and distilled water for 5 minutes. (x) Stained washed sections were mounted with glycerol gelatin (aqueous-based mounting medium, Aquaperm™, Thermo Shandon). Parallel negative (tissues from healthy goat) and positive (previously confirmed by RT PCR and histopathology) controls were run each time.

### Reverse transcription - Polymerase chain Reaction

RNA was extracted from lymphnode tissue homogenates using RNeasy Kit (Qiagen, Germany) following the procedure described by the manufacturer. Reverse transcription-polymerase chain reaction (RT-PCR) was carried out with F and N gene-specific primer sets of PPRV as described by Forsyth and Barrett [22] and Qiagen one step RT-PCR kit. The pieces of smeared filter papers were directly used in PCR tubes as source of template RNA. The RT-PCR technique was used as described by Michaud et al. [30].

### Sequencing and Analysis of nucleotide and deduced amino acid sequence data

One of the amplified RT-PCR products were sequenced directly using PCR primer specific for F and N genes as used before. The PCR products for both the genes were cleaned using the EZ-10 Spin column DNA Gel extraction kit (Bio Basic Inc., USA). The procedure was strictly followed as described by the manufacturer. Quantification of purified DNA, cycle amplification and sequencing of the products were done from 1st BASE Laboratories, Singapore. The nucleic acid sequences obtained from PCR products of F and N gene and their deduced amino acid sequences were aligned and studied for their divergence. The sequences of the isolate have been submitted to the GenBank. Homology and divergence among the Bangladeshi isolates have been studied. Sequenced data of Bangladeshi isolates were compared with other related sequences retrieved from the GenBank. Sequence editing, alignment, and homology study were carried out with the software package “Lasergene” (Modules - EditSeq and MegAlign; DNASTAR Inc., USA).

### Ethical approval

The present study was not subject to ethical approval as Bangladesh laws don’t require approval for studies not involving experimental inoculation. The samples originated from natural outbreaks and from dead animals. The nasal swabs were collected from sick animals by registered veterinarian. The post mortem was conducted by registered veterinarian as per country rule.
